# A Partially Distributed Intrusion Detection System for Wireless Sensor Networks

**DOI:** 10.3390/s131215863

**Published:** 2013-11-25

**Authors:** Eung Jun Cho, Choong Seon Hong, Sungwon Lee, Seokhee Jeon

**Affiliations:** Department of Computer Engineering, Kyung Hee University, Gyeonggi-do 446-701, Korea; E-Mails: d2o2mask@khu.ac.kr (E.J.C.); drsungwon@khu.ac.kr (S.L.)

**Keywords:** Bloom filter, intrusion detection system, low memory usage, wireless sensor network, security

## Abstract

The increasing use of wireless sensor networks, which normally comprise several very small sensor nodes, makes their security an increasingly important issue. They can be practically and efficiently secured using intrusion detection systems. Conventional security mechanisms are not usually applicable due to the sensor nodes having limitations of computational power, memory capacity, and battery power. Therefore, specific security systems should be designed to function under constraints of energy or memory. A partially distributed intrusion detection system with low memory and power demands is proposed here. It employs a Bloom filter, which allows reduced signature code size. Multiple Bloom filters can be combined to reduce the signature code for each Bloom filter array. The mechanism could then cope with potential denial of service attacks, unlike many previous detection systems with Bloom filters. The mechanism was evaluated and validated through analysis and simulation.

## 1. Introduction

Wireless sensor networks (WSNs) are composed of several very small sensor nodes. They are generally designed to operate in non-infrastructure environments using inexpensive hardware. This can be achieved using IEEE 802.15.4 PHY/MAC [1] and the ZigBee [2] stack. Sensor nodes can be assigned IPv6 addresses using IPv6 over Low-power Wireless Personal Area Networks (6LoWPAN) [3]. An Internet of Things [4] can be achieved using WSNs. However, security vulnerabilities associated with the IP networks in the WSNs may result. The wide potential applicability of WSNs, including in critical infrastructure, makes their security a prominent concern.

Intrusion detection systems (IDSs) are practical and efficient security mechanisms. They can be classified into two categories by their method of attack detection: those which use attack signatures and those which use abnormal states. The latter type requires large amounts of memory to store the traffic states and high computation power to analyze traffic, making them unsuitable for use in sensor nodes. Signature-based IDSs require the storage of many attack signatures, which increases memory use. A behavior-based IDS needs strong processing power to analyze abnormal behavior. While conventional IP networks can employ various intrusion detection mechanisms, the high resource requirements make some systems less suited to wireless sensor nodes, and several researchers have sought to develop various systems compatible with WSNs [5]. Most such IDSs analyze anomalous behavior; signature-based IDSs for WSNs receive much less attention. Syed *et al.* [6] proposed an IDS based on Bloom filter arrays for implement on WSNs. Their approach significantly reduces the size of attack signatures. This paper analyzes the threats inherent to this previous work, proposes a partially distributed IDS mechanism to overcome these threats, and optimizes memory and energy usage.

This paper is organized as follows: Section 2 describes the basic concept of a Bloom filter. Section 3 describes the problems of the previous system and proposes a partially distributed IDS mechanism. Section 4 tests our proposal through mathematical analysis. Finally, concluding remarks are presented in Section 5.

## 2. Related Work

### 2.1. Bloom Filter

Bloom filters can efficiently reduce the data structure size [7] by using multiple hash functions. A Bloom filter has a specific false positive rate based on the number of hash functions, the amount of input data, and the size of the Bloom filter array. The characteristics of Bloom filters and their applications are described in detail elsewhere [7]. The false positive rate can be controlled by optimizing the size of the Bloom filter array.

**Figure 1.**
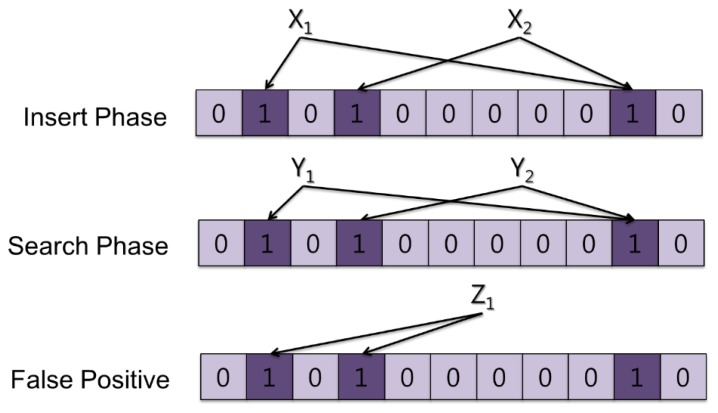
Simple operation of a Bloom filter.

Figure 1 shows a basic example of Bloom filter operation. All the Bloom filter array spaces are initialized at 0 before they are used. During the Insert phase, input data points X_1_ and X_2_ are passed through multiple hash functions. The Bloom filter in Figure 1 has two hash functions and produces two results that indicate the specific positions of each input data point. In the figure, X_1_ indicates positions 2 and 10. Each bit of the corresponding positions is set to 1. X_2_ indicates the positions 4 and 10. These processes occur whenever data are inserted into the Bloom filter array during the Insert phase. After the Insert phase, the Bloom filter arrays are implemented in the specific system to be used. During the Search Phase, data represented by Y_1_ and Y_2_ arrive at the system with the Bloom filters. Once the data are passed to the two hash functions used in the Insert phase, two results are derived. If the corresponding position bits are 1, the inserted data point matches the Bloom filter. Figure 1 shows that Y_1_ derives positions 2 and 10, with both position bits already marked as 1. Therefore, we can say that the data Y_1_ was inserted as input data during the Insert phase. However, some other data (such as Z_1_ in Figure 1) might give a false positive result. When Z_1_ passes through the hash functions it derives positions 2 and 4, both of which have a value of 1. Therefore, it may mistakenly be concluded that Z_1_ was inserted during the Insert phase. The combination of the two data points causes Z_1_ to match to the Bloom filter because the bit at position 2 is marked by X_1_ and that at position 4 is marked by X_2_. Therefore, a false positive occurs. If more data are inserted into the Bloom filter array, the false positive rate will increase. However, if more space is available for the array, the false positive rate will decrease.

Syed *et al.* [6] reported the expected false positive rate *fpr* to be calculated as follows:

(1) $$\text{fpr}={\left(1-{\left(1-\frac{1}{m}\right)}^{kn}\right)}^{k}\approx {\left(1-e^{\overset{-kn}{\;}/\underset{m}{\;}}\right)}^{k},$$

where *n* is the number of input data points, *m* the array size of the Bloom filter, and *k* the number of hash functions. Equation (2) can be derived for the simple case of two hash functions (*i.e.*, *k* = 2) [7]:

(2) $$m\geq n\log_{2}e\log_{2}\;\left(\overset{1}{\;}/\underset{\text{fpr}}{\;}\right)\approx 1.44n\log_{2}\;\left(\overset{1}{\;}/\underset{\text{fpr}}{\;}\right).$$

This equation allows the minimum memory requirements needed to achieve a specific *fpr* to be calculated for other given variables.

### 2.2. 6LoWPAN

6LoWAPN [3] is an Internet Engineering Task Force (IETF) working group that defines an encapsulation and header compression mechanism to assign IPv6 addresses to small devices using IEEE 802.15.4 [1], which supports low-rate wireless personal area networks. IEEE 802.15.4 has the capability to deliver packets of up to 127 bytes. However, many bytes are consumed by overhead in 6LoWPAN. At the application layer, only 33 bytes of free space is available in the worst case, meaning that data over 33 bytes should be segmented.

### 2.3. IDSs using Bloom Filters

Syed *et al.* [6] proposed an IDS for a WSN using a Bloom filter. In a WSN, the resource-constrained sensor nodes require mechanisms implemented in them to be resource-efficient. A sensor node uses much more energy when sending and receiving data than when performing complex calculations. Therefore, mechanisms for WSNs must be designed with reduced messaging overhead. Syed *et al.* [6] used Bloom filters to reduce the memory usage of IDSs based on signatures. The application of a complete set of SNORT [8] rules, including 13,339 attack patterns, uses about 252 KB of memory. However, using a Bloom filter in the IDS reduces memory use by 13 KB when there are two hash functions and *fpr* = 0.024.

**Figure 2.**
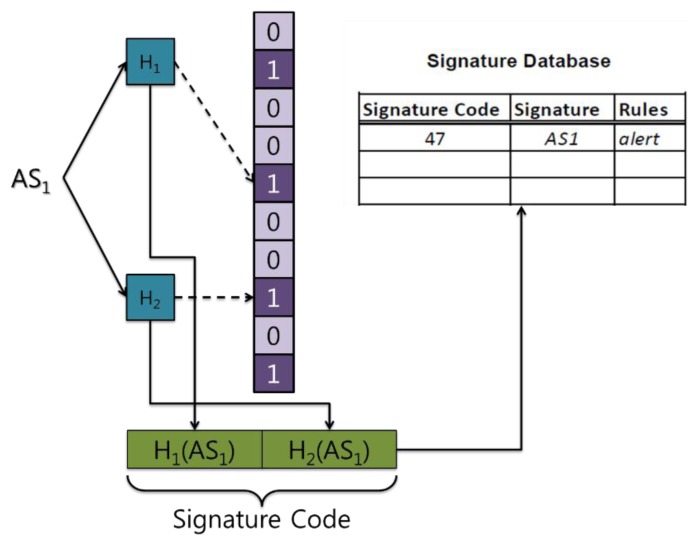
IDS operation using Bloom filters.

When a specific attack is detected, action needs to be taken against it. A correct response requires knowledge of the kind of attack. However, attacks cannot be identified using a Bloom filter array. To overcome this, Syed *et al.* [6] proposed generating a signature code that is then sent to the gateway (or sink) where the signature database resides (Figure 2). There are corresponding rules in the signature database that determine which action to take against a specific attack.

## 3. Problem Statement and Proposal

### 3.1. Problem Statement

Syed *et al.* [6] used a Bloom filter to reduce the signature size. When an attack is detected, the sensor node sends the signature code to the gateway to identify the attack. However, the attacker can instruct sensor nodes to send useless messages to the gateway because every Bloom filter has a certain *fpr*. Sensor nodes are usually deployed in an open area, and thus an attacker may be able to access them and extract information regarding the Bloom filter array. If an attacker can identify false positive messages to the Bloom filter array in the sensor nodes, he would be able to send repeated messages to several sensor nodes. The sensor nodes receiving such messages would check them and identify malicious messages. The sensor nodes would then send the signature code to the gateway. From the gateway's perspective, when many sensor nodes begin to send a signature code, it would not be identified as an attack signature code. This functions as a Denial of Service (DoS) attack on the gateway. In this situation, sensor nodes will exhaust their limited battery power.

### 3.2. Partially Distributed IDS Mechanisms

Our previous work proposed a distributed IDS mechanism to address the above problems [9]. However, the proposal had a limitation: relay nodes participate in an IDS on the network layer, causing the distributed IDS mechanism to operate on the network layer, which leads to the module not being able to detect fragmented attack signatures. To improve the distributed IDS and to address the problem described above, we extend our previous work and evaluate our new proposal in terms of power consumption, memory use, and detection rate.

The goals of this work are to develop a system that can:

- Detect fragmented attack packets;
- Use less memory and energy than the system of Syed *et al.* [6].

To solve the problem encountered by [9] and to enhance resource efficiency, we propose a partially distributed IDS using multiple Bloom filter arrays. The assumptions of our proposal are:

- The wireless sensor network works in an *ad hoc* manner;
- Multiple Bloom filter arrays are randomly deployed;
- Every node has two types of Bloom filter array.

#### 3.2.1. Insert Phase for the Partially Distributed IDS

To construct a partially distributed IDS, we propose a classification method to distribute attack signatures among multiple Bloom filter arrays. As mentioned above, 6LoWPAN has 33 bytes of space for the application layer in a packet. Data over the 33 byte limit should be fragmented. This can cause problems, because if the attack signature is fragmented into several packets, the IDS working on the network layer will not be able to detect it. In SNORT V2.8 [8], the maximum length of an attack signature is 487 bytes; the average length is 20 bytes [6]. The string length distribution of SNORT V2.3.2 has been reported previously [10]. These statistics indicate that the average size of an attack signature using the SNORT rule sets will be less than 33 bytes, and hence these attack signatures do not need to be fragmented.

However, the fragmenting of attack signatures into several packets generally cannot be avoided due to their size. To detect fragmented attack signatures, multiple Bloom filter arrays are used in the application layer and the network layer.

During the Insert phase, two kinds of Bloom filter are made: one for the application layer to detect fragmented attack signatures and one for the network layer to detect unfragmented attack signatures. To detect fragmented attack signatures, data should be reassembled by the IDS. If fragmented packets are reassembled whenever the IDS checks them, processing power will be wasted. Moreover, the relay nodes should retain the fragmented packets until all its parts have arrived. This significantly increases the end-to-end delay. In this paper, we propose a multiple-layer IDS to efficiently detect the attack signatures. The IDS can detect fragmented attack signatures at the application layer and can detect unfragmented attack signatures at the network layer.

An application layer IDS does not need a new mechanism to make a Bloom filter array. Every sensor node has the same Bloom filter array, and these can detect all attack signatures that are more than 33 bytes long. However, at the network layer, the IDS uses a distributed IDS mechanism to reduce memory and energy use.

**Figure 3.**
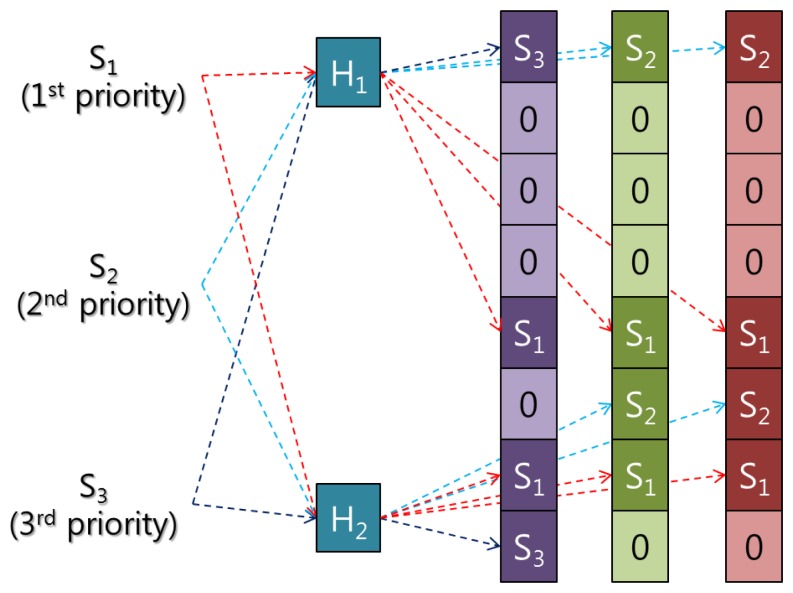
Simple example of the Insert phase of an IDS in the network layer.

Attack signatures over 33 bytes are passed through hash functions and marked on the application-layer Bloom filter array. However, another mechanism is needed for the network layer to enhance the detection rate and to reduce the memory use of the IDS. WSNs generally work in an ad hoc manner with nodes acting as end points and intermediate nodes. In this case, intermediate nodes, which are also called relay nodes, can participate in intrusion detection. To distribute the attack signatures into several Bloom filter arrays, we use the priority feature of the SNORT rule set. Figure 3 shows a simple example of the Insert phase of a Bloom filter array in the network layer. In the figure, S_1_, S_2_, and S_3_ represent signatures 1, 2, and 3, respectively.

In Figure 3, S1 has the highest priority, and S3 the lowest. Without the priority feature of SNORT, S_1_, S_2_, and S_3_ would be stored in only one Bloom filter array, and would have the same detection rate. However, the SNORT rule prioritizes attack signatures, defining as first priority those attack signatures that can cause serious problems to the system, and as third priority attack signatures that are merely unusual traffic. As such, if a first-priority attack signature is missed, the system could be disabled. To prevent this, the detection rate of first-priority attack signatures must be increased. S_1_, which has the highest priority, can be stored in every Bloom filter array, while S3 is stored in only one Bloom filter (Figure 3). Higher-priority signatures can be stored in more Bloom filters compared with those with lower priorities. This distribution mechanism increases the detection rate of the higher-priority signatures, without compromising the detection rates of lower-priority signatures. Algorithm 1 shows the process of creating the proposed Bloom filter arrays.

**Algorithm 1.** Insert phase of Bloom filter arrays.
**for** signature *i* = 1 : *n*
**do** Hash the signature *i* using two hash function  **if** signature *i* is lower than 33 bytes **then** **if** signature *i* has 1st priority **then**    insert hashed signature *i* into all BF arrays   **else if** signature *i* has 2nd priority **then**    insert hashed signature *i* into half of BF arrays   **else**    insert hashed signature *i* into a BF array   **end if**  **else**    insert hashed signature *i* into an application layer BF array   **end if**   **end for**


#### 3.2.2. Operation of Partially Distributed IDS

A node can communicate directly with its neighboring nodes at a distance of one hop. Relay nodes are required for more distant nodes to communicate, because ad hoc networks have no infrastructure support for nodes. This means that a node is not only the end point of communication but also a relay node for the delivery of data packets. A packet may pass through many relay nodes to arrive at its destination node. When packets pass through relay nodes, the relay nodes can detect attack signatures in them using the IDS in the network layer. If the packet is fragmented, the attack signature will be divided into several packets, and relay nodes would not be able to detect attack signatures. At the receiver, the fragmented packet will be reassembled and the attack signature would be detectable at the application layer.

**Figure 4.**
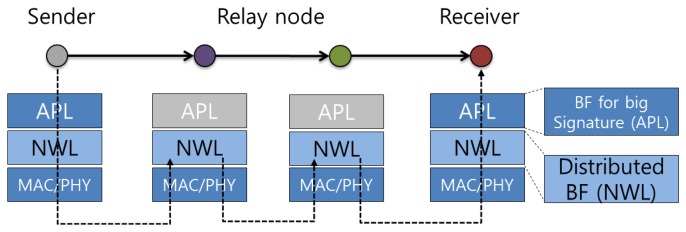
Example of the proposed IDS operation.

**Figure 5.**
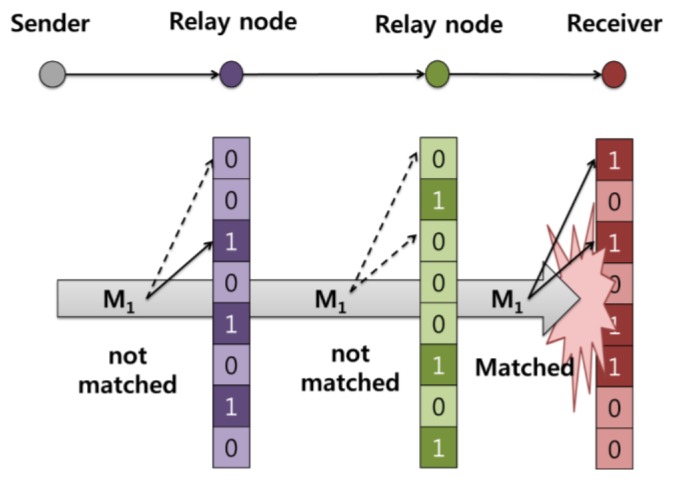
Operation of the distributed IDS on the network layer.

Figure 4 shows how relay nodes participate in this partially distributed IDS mechanism. At the relay node, only the network layer IDS can participate in intrusion detection. Figure 5 shows in detail the operation of the distributed IDS on the network layer: a packet *M1* is sent by the sender to the receiver, with two relay nodes aiding the delivery. When the packet arrives at a relay node, the node uses its own Bloom filter array in the network layer to check the packet. Figure 5 shows packet *M1* passing two relay nodes without matching; however, a match is found at the receiver, and an attack signature is detected. In our proposed system, relay nodes maintain databases of different combinations of attack signatures. This means that the node does not need to store all the attack signatures by itself. Based on Equation (2), our mechanism can reduce memory use compared with the previously described system [6].

## 4. Performance Analysis

This section analyzes the energy and memory used by the proposed method, along with its detection rate. The energy and memory use are compared against those reported for the mechanism of Syed *et al.* [6].

### 4.1. Memory Use

Section 2.1 states that the minimum memory requirements can be estimated for a specific *fpr*; *i.e.*, the memory required depends on the amount of input data. If the amount of input data decreases, the memory used by the Bloom filter arrays can be reduced. Therefore, a specific *fpr* can be obtained by reducing the size of the Bloom filter array. However, to achieve a similar *fpr* to that reported in the previous work [6], we need to adjust the *fpr* based on the number of distributed IDSs, because all data points have an independent *fpr* at each Bloom filter array. For the remaining *fpr* in the same level, the *fpr* value should be divided by the number of distributed IDSs when multiple Bloom filter arrays are implemented. The size of the Bloom filter array in the application layer also needs to be determined to give the total memory use. This leads to Equation (3) from the previous work [6], which computes the minimum memory required for the distributed IDS based on the number of Bloom filter arrays *j*:

(3) $$m\geq 1.44\frac{n}{j}{\text{log}}_{2}\left(\frac{j}{\text{fpr}}\right)$$

Equation (3) can be used to calculate the memory requirements of the network layer IDS. Calculation of the total memory requirement must include the memory requirement of the application layer IDS. Equation (4) can be derived from Equation (3):

(4) $$m\geq 1.44\left(1-k\right)n{\text{log}}_{2}\frac{1}{\text{fpr}}+1.44\frac{nk}{j}{\text{log}}_{2}\left(\frac{j}{\text{fpr}}\right)$$

where *k* is the ratio of attack signatures of less than 33 bytes, *m* the size of the Bloom filter array of a node, and *n* the total number of attack signatures. This equation can be modified to incorporate the priority-based distribution mechanism of Section 3.2.1, where *a*, *b*, and *c* are ratios of priority 1, 2, and 3 in SNORT, respectively:

(5) $$m\geq 1.44\left(1-k\right)n{\text{log}}_{2}\frac{1}{\text{fpr}}+1.44nk\;\left(a+\frac{b}{2}+\frac{c}{j}\right)\;{\text{log}}_{2}\;\left(\frac{j}{\text{fpr}}\right)$$

To reduce the number of messages, the signature database and response policy need to be stored in sensor nodes. An attack signature can be compressed using hash functions; it can be reduced to 4 bytes if two hash functions are used and if there are fewer than 65,536 input data points [5]. A single byte can be used to express 256 kinds of policy; therefore, 5 bytes per attack signature is required to create the signature database. Assuming that Equation (3) is *M*, the total memory requirements, including the signature database, can be calculated as shown in Equation (6):

(6) $$m\geq M+40n\;\left\{\left(1-k\right)+\left(a+\frac{b}{2}+\frac{c}{j}\right)\;k\right\}$$

**Figure 6.**
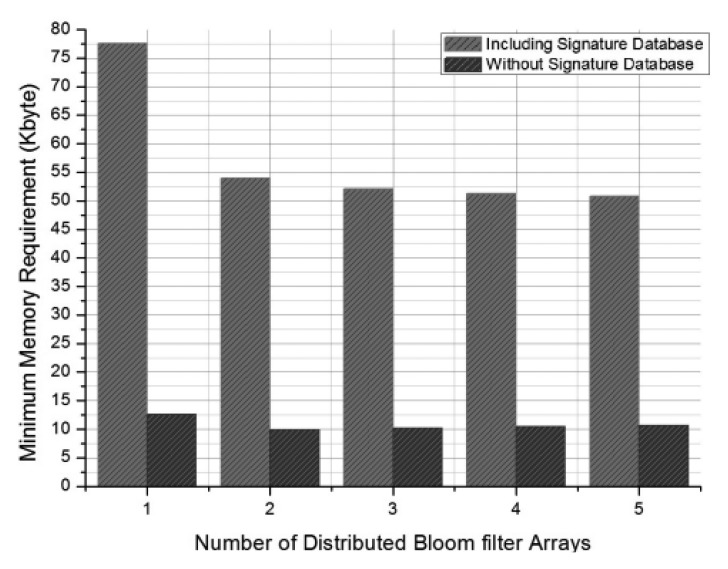
Minimum memory requirements according to the number of distributed Bloom filter arrays *j*.

Calculations in this paper use the parameters of Syed *et al.* [6] to compare the two methods' memory and energy use. Syed *et al.* [6] had an *fpr* of 0.024 and used 13,312 attack signatures. SNORT can be used to determine the ratio of attack signatures that are of less than 33 bytes. An approximation of *k* = 0.92 can be obtained, and *a*, *b*, and *c* are set as 0.3, 0.48, and 0.22, respectively, according to the *classification.conf* file included in SNORT. Figure 6 compares the minimum memory requirements of the previous method [6] and of the proposed method according to the number of distributed Bloom filter arrays *j*.

When *j* = 1, the Bloom filter array is not distributed, and the two methods are equivalent. The figure shows that memory use can be reduced by 30% when two distributed Bloom filter arrays are used instead of one. Our method uses more memory space than the previous method [6] does, but as a tradeoff it allows the storage of the signature database on the sensor nodes. This design eliminates overhead messages that can allow potential DoS attacks. A memory saving of 10% compared with the previous method [6] can be achieved if the proposed method is implemented without a signature database.

Memory use by the proposed system was also compared with that of a completely distributed IDS [9]. The completely distributed system can be simulated by setting *k* = 1 in Equation (5), which represents that no attack signatures are to be fragmented. Our method uses more memory space than the completely distributed system. For example, when *k* = 0.9, our system uses 9.7 KB of memory to store the Bloom filter array *vs.* 9.4 KB used by the completely distributed system. Our proposed system uses two Bloom filter arrays for each layer, and all the sensor nodes have the same Bloom filter array in an application layer. Unfragmented attack signatures can be detected at an application layer, but this requires all unfragmented attack signatures to be inserted into a single Bloom filter array operated on the application layer. This increases the memory required by the proposed system compared with that used by the completely distributed IDS [9]. As such, there is a tradeoff between detection efficiency and memory use.

### 4.2. Energy Consumption

The specifications of CC2430 [11] and Hmote 2430 [12] sensor nodes are used to compare the energy consumption by our proposed method and by the previous system [6]. The Hmote 2430 (Hybus, Korea) uses CC2430 chips for its micro control unit (MCU) and transceiver. 6LoWPAN (named in the *Nanostack*) can be implemented on the Hmote 2430 using FreeRTOS [13]. The CC2430 chip uses 24.7 mA and 27 mA for sending and receiving, respectively [12]. When it uses the MCU at its highest speed, it uses 7.0 mA [12]. The amount of energy needed to transfer and receive specific packets can be calculated almost exactly for a given transfer rate. For example, the transfer of 1,024 Kbit at 128 kbps will take 8 s. The total energy consumption can be calculated from the required time and the rate of energy consumption. However, the exact running time of the hash function cannot be estimated; it can be measured using the Hmote 2430 node, which can also measure the data transfer. Running times of a simple integer hash function and a data transfer program are found to be 6 ms and 120 ms (average, range 87–187 ms), respectively. The average time for data transfer is used to calculate the energy consumption. Table 1 lists the symbols used in the following calculations of the expected energy consumption:

(7) $${\text{Energy}}_{\text{conv}}=\left(q+\left(p-q\right)\text{fpr}\right)hs+pc$$

(8) $${\text{Energy}}_{\text{prop}}=\text{phc}$$

where *c* and *s* are constants (*c* = 0.03442 w and *s =* 0.000126 w, as measured during testing). Because data can be transferred and received simultaneously, the energy consumption by both processes is summed in calculating *c*. The previous IDS [6] checks each packet when it arrives at the destination; therefore, the hash function is run only once. However, whenever the sensor nodes detect a suspected attack signature (whether true or not), they must send 4 bytes of signature code to the signature database. This increases the energy consumption, and thus introduces a potential vulnerability to DoS attacks.

**Figure 7.**
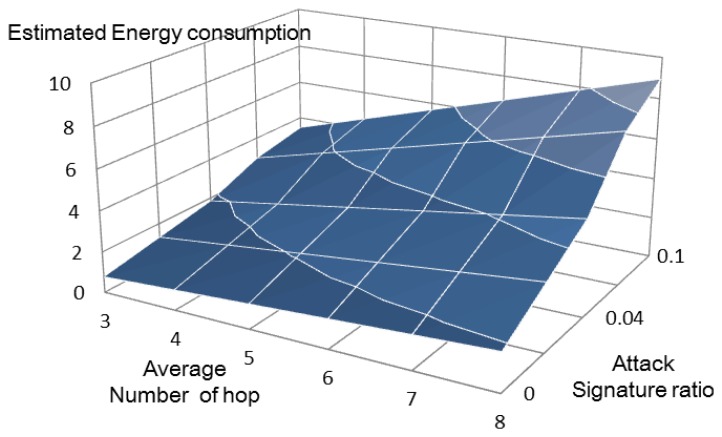
Estimated energy consumption of the previous method.

**Figure 8.**
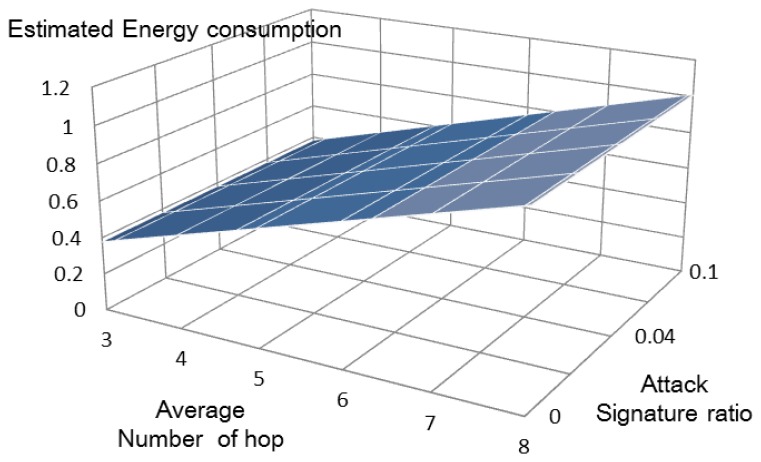
Estimated energy consumption of the proposed method.

The total energy consumptions of the previous method [6] (Figure 7) and our proposed method (Figure 8) can be estimated using Equations (7) and (8), respectively.

**Figure 9.**
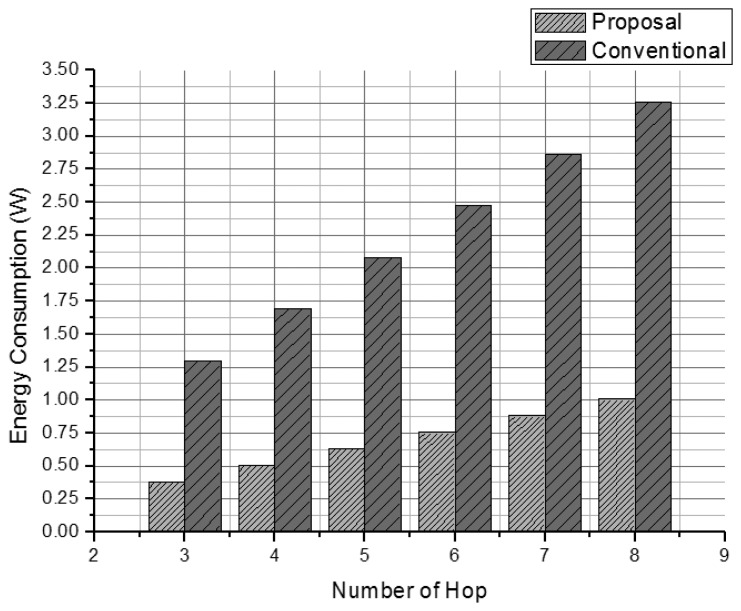
Estimated energy consumption.

Our method does not show increased energy consumption as the number of attack signatures increases. By comparison, the previous method [6] shows rapidly increasing energy consumption under similar conditions. Figure 9 compares the energy consumed by the two systems. Our proposal uses about 20% of the energy used by the other system, because it eliminates the overhead messages.

### 4.3. Detection Rate

There is no detection rate in signature-based IDS. If a system includes more attack signatures, more attacks can be detected, and if a system does not include a specific attack signature, it cannot detect that attack at all. However, our proposal does have a detection rate, despite it being a signature-based IDS. The proposed mechanism distributes attack signatures into several Bloom filter arrays. Several nodes help to deliver a packet as it is passed along a specific path. If all the relay nodes have the same Bloom filter array, the probability that attack packets can be detected is only $\left(a+\frac{b}{2}+\frac{c}{j}\right)$ in the worst case. If the hop distance to deliver the packet is longer, the detection probability increases.

The detection rate *D* of our proposal can be calculated probabilistically (Equation (9)) based on the number of Bloom filter arrays *j*, the average number of hops *h*, and the ratio of attack signatures shorter than 33 bytes *k*:

(9) $$D=\left(1-k\right)+\sum_{z=0}^{h-1}{\left(1-\frac{1}{j}\right)}^{z}\frac{1}{j}$$

**Figure 10.**
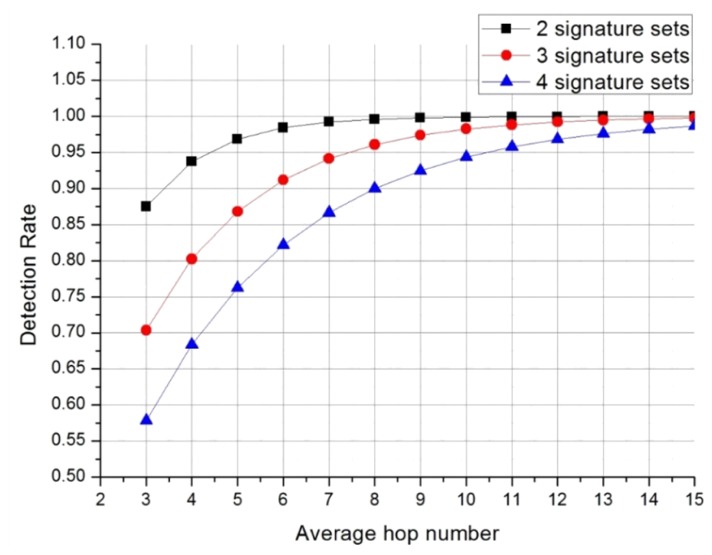
Detection rates when attack signatures are distributed.

The detection rate of an application layer IDS is (1 − *k*). An application layer IDS can detect all attacks that are stored in its application layer Bloom filter array owing to it not being a distributed system. At the network layer, the IDS has a distributed Bloom filter array, which cannot detect all kinds of attack signatures. Hence, the detection rate of a network layer IDS has to be calculated. The calculation of the detection rate at a specific hop number is a simple probability problem. For example, two probabilities should be considered for assessing detection of an attack signature on the second hop. The first is the undetected rate of an attack signature on the first hop (*i.e.*, (1 − (1/*j*))). For an attack signature to be detected on the second hop, it cannot have been detected on the first hop. The second probability is the detection rate of the attack signature on the second hop. The detection rate on a specific hop is 1/*j*. The total detection rate for two-hop system is the product of these two probabilities. Equation (9) follows simply for the calculation of the total detection rate of a network layer IDS. Figure 10 shows detection rates obtained from Equation (9).

Detection rates of around 95% are achieved by systems with averages of 4–10 hops. From a practical viewpoint, the highest efficiency is achieved using two Bloom filter arrays. However, Equation (9) cannot be applied to the priority-based distribution mechanism described in Section 3.2.1. If *a*, *b*, and *c* are the first, second, and third priority SNORT rules, respectively, then Equation (10) can be derived from (9):

(10) $$\text{D}\prime =1-k+a+b\sum_{z=0}^{h-1}{\left(1-\frac{1}{2}\right)}^{z}\frac{1}{2}+c\sum_{z=0}^{h-1}{\left(1-\frac{1}{j}\right)}^{z}\frac{1}{j}$$

**Figure 11.**
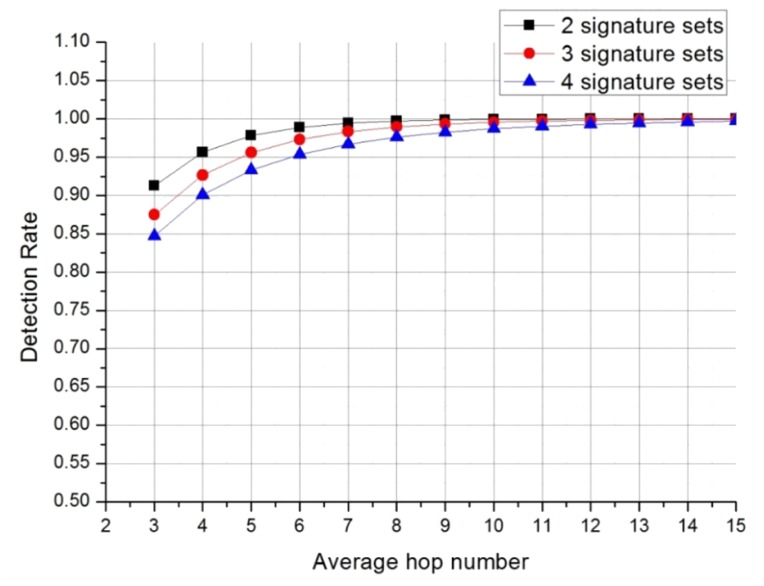
Detection rates when attack signatures are distributed by priority.

The detection rates shown in Figure 11 are calculated for *a*, *b*, and *c* being 0.3, 0.48, and 0.22, respectively, as obtained from the *classification.conf* file included in SNORT.

A comparison of Figures 10 and 11 shows that detection is better when a priority-based distribution mechanism is applied. An average hop distance of 6 leads to 95% detection rate being achieved with four Bloom filter arrays (Figure 11). When two Bloom filter arrays are used, over 98% can be achieved with a 6-hop distance. Similar 95% detection is achieved in the non-prioritized system using four Bloom filter arrays under similar conditions with an average hop distance of 10 (Figure 10).

**Figure 12.**
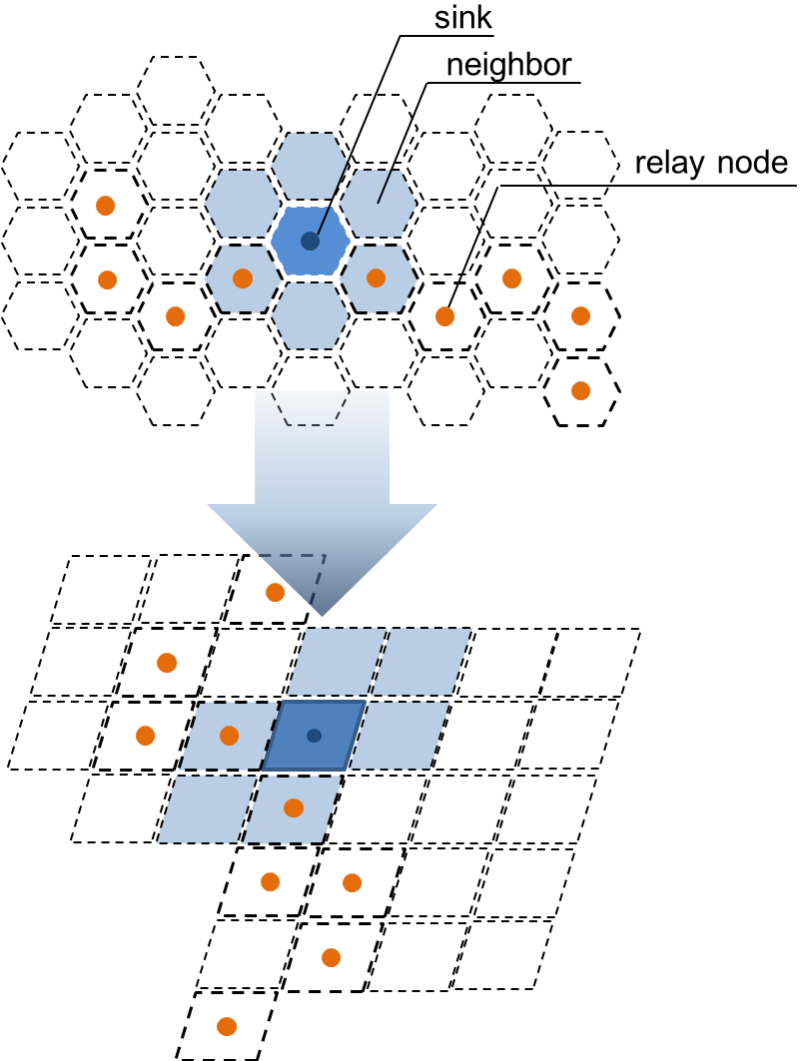
Simulation model and expression in C++.

The above results are based on calculations. In a network environment, the network topology also needs to be considered. To apply the network topology, C++ simulations that can measure detection rates are performed. We model the hexagonal direction space for each sensor node (Figure 12), and each sensor node has one distributed Bloom filter array.

**Figure 13.**
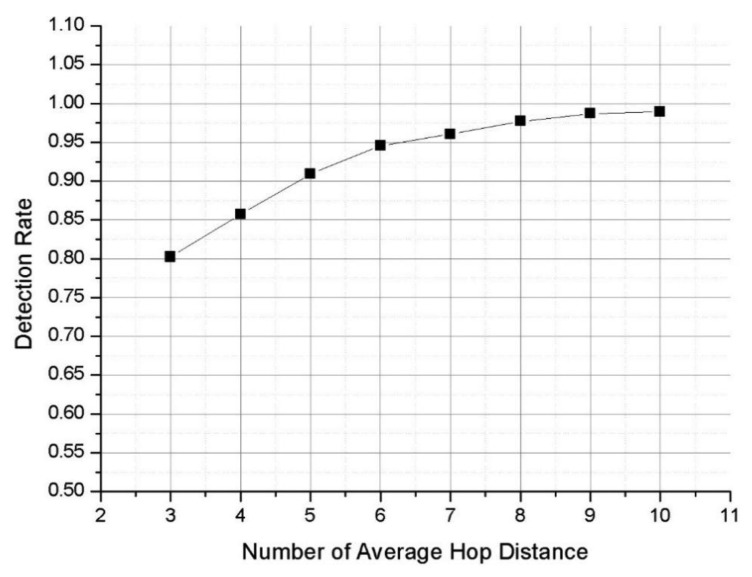
Simulated detection rates.

The frequency of attack signature statistics from Ahnlab [14] are used to generate the attack signature codes used in the simulation (Table 2). The attack signatures are distributed into the three Bloom filter arrays based on our proposed method. The results show that the detection rate increases with increasing average hop distance (Figure 13).

When compared with the analysis (Figure 11), the simulation (Figure 13) finds a lower detection rate. In the simulation, attack signatures are generated in set proportions (Table 2), and the analysis uses a slightly different distribution of attack signatures.

The simulation considers the real network topology shown in Figure 12, while the mathematical analysis cannot consider network topology. These factors resulted in the different detection rates shown in the two figures. However, in both cases, detection rates increased with increasing average hop distance. The analysis and the simulation both show that our proposed system works well when WSNs have larger hop distances.

## 5. Conclusions

We propose here a partially distributed IDS for WSNs. Distributing attack signatures into several Bloom filter arrays can reduce memory and energy requirements. Our proposed method also eliminates overhead messages that can account for significant energy consumption. Analysis demonstrated that the method uses less energy and memory than reported for a previous system [6]. The proposed system can also prevent DoS attacks. Its multi-layer mechanism can detect fragmented attack signatures, whereas a completely distributed IDS [9] cannot. Specific requirements might depend on a system's purpose: some WSNs require very low memory use, others require high detection rates. The proposed system can control these factors based on specific requirements. To minimize memory use, more distributed Bloom filter arrays can be used; to maximize the detection rate, fewer Bloom filter arrays can be used. Our proposed method can add flexibility to the operation of an IDS in a WSN.

## Figures and Tables

**Table 1.** Symbols used in energy consumption calculations.

**Symbol**	**Description**
*c*	Amount of energy consumption for running hash functions
*s*	Amount of energy consumption for signature code transfer
*h*	Average distance of packet delivered
*p*	Total number of packets
*q*	Number of attack packets

**Table 2.** Attack signatures, their occurrence, and priority from Ahnlab.

**Rank**	**Name**	**Percentage**	**Priority**
1	Win-Trojan/downloader	10.4	3
2	Swf/Agent	9.2	2
3	Win-Trojan/Agent	9.1	1
4	Win-Adware/Korad	7.9	1
5	JS/Agent	7.4	2
6	Textimage/Autorun	7	1
7	Win-Trojan/Onlinegamehack	5.5	1
8	JS/Exploit	5	3
9	Html/Agent	4.9	2
10	JS/Iframe	4.6	3
11	Win32/Virut	4.3	2
12	Swf/Cve-2011-2110	4.1	1
13	Win32/Conflicker	3.6	2
14	Swf/Exploit	3	2
15	Win32/Autorun.worm	3	1
16	Win-Trojan/Startpage	2.6	3
17	Win-Trojan/Winsoft	2.3	1
18	Win32/Kido	2.3	1
19	Dropper/Malware	2.1	1
20	Win-Downloader/Korad	1.9	2
